# Polymorphisms in *CFI* and *ARMS* genes and exudative age-related macular degeneration: Correspondence

**DOI:** 10.22099/mbrc.2022.44737.1782

**Published:** 2022

**Authors:** Rujittika Mungmunpuntipantip, Viroj Wiwanitkit

**Affiliations:** 1 Private Academic Consultant, Bangkok Thailand; 2 Honorary professor, Dr DY Patil University, Pune, India

*Dear Editor,*

We would like to share ideas on the publication “Characterization of polymorphisms in *CFI* and *ARMS* genes and their association with exudative age-related macular degeneration in Algerian patients [1].” According to Abid et al., this work enriches the bank of data pertaining to the *CFI* and *ARMS2* genes by disclosing for the first time the allelic and genotypic frequencies of these genes' polymorphisms that define the Algerian population [1]. Exudative age-related macular degeneration might or might not be impacted by the hereditary component. The effect of a polymorphism is investigated in this study. We both concur that exudative age-associated macular degeneration may be related to the underlying genetic component under research. However, exudative age-related macular degeneration has been linked to numerous genetic variations. Examples of gene polymorphisms include selectin and tumor necrosis factor-alpha polymorphisms [2, 3]. Future research should examine the implications of unexpected, possibly perplexing genetic variants.

## Conflict of Interest:

None
